# Nociception in fruit fly larvae

**DOI:** 10.3389/fpain.2023.1076017

**Published:** 2023-03-17

**Authors:** Jean-Christophe Boivin, Jiayi Zhu, Tomoko Ohyama

**Affiliations:** ^1^ Department of Biology, McGill University, Montreal, QC, Canada; ^2^ Integrated Program in Neuroscience, McGill University, Montreal, QC, Canada; ^3^ Alan Edwards Centre for Research on Pain, McGill University, Montreal, QC, Canada

**Keywords:** *Drosophila*, nociception, behavior analysis, connectome, neural circuit, neuromodulation

## Abstract

Nociception, the process of encoding and processing noxious or painful stimuli, allows animals to detect and avoid or escape from potentially life-threatening stimuli. Here, we provide a brief overview of recent technical developments and studies that have advanced our understanding of the *Drosophila* larval nociceptive circuit and demonstrated its potential as a model system to elucidate the mechanistic basis of nociception. The nervous system of a *Drosophila* larva contains roughly 15,000 neurons, which allows for reconstructing the connectivity among them directly by transmission electron microscopy. In addition, the availability of genetic tools for manipulating the activity of individual neurons and recent advances in computational and high-throughput behavior analysis methods have facilitated the identification of a neural circuit underlying a characteristic nocifensive behavior. We also discuss how neuromodulators may play a key role in modulating the nociceptive circuit and behavioral output. A detailed understanding of the structure and function of *Drosophila* larval nociceptive neural circuit could provide insights into the organization and operation of pain circuits in mammals and generate new knowledge to advance the development of treatment options for pain in humans.

## 1. Introduction

Nociception refers to the process by which an animal's nervous system encodes actual or impending tissue damage (1). It is crucial for survival, as it prompts the animal to react in a way that minimizes further harm. It is thought that nociception evolved about 550 million years ago and that the subjective experience of pain is a key manifestation of this process (2). It is well-established that activation of nociceptors and nociceptive pathways can give rise to pain in humans, and that activation of comparable receptors and pathways in animals can trigger behaviors suggestive of pain perception in animals as well. Until methods to assess subjective pain in animals are developed, however, it will remain difficult to assess whether pain can be dissociated from nociception as in humans (1). In the meantime, animal models continue to offer the advantage of yielding mechanistic insights into how nociceptive neurons activate behaviors that minimize actual or impending harm. In particular, investigations of nociceptively induced behaviors that can be measured with high-throughput methods in the fruit fly, which have tractable nervous systems that are readily imaged and manipulated with a multitude of genetic techniques, have the potential to illuminate in detail how networks of neurons interact to process nociceptive inputs and generate behaviors that appropriately minimize harm and improve survival.

Although fruit flies are phylogenetically distant from mammals, numerous studies have shown that receptors expressed in their nociceptive sensory neurons are evolutionarily conserved. Several ion channels, including transient receptor potential A (TrpA), degenerin/epithelial sodium (DEG/ENaC), calcium channel subunit α2δ3, Piezo, and L-type voltage-gated calcium channel (L-VGCC) all play important roles in both fly and mammalian nociception (3–9). For example, familial episodic pain syndrome (FEPS), a rare genetic form of peripheral neuropathy, results from a missense mutation that alters a single amino acid of the hTRPA1 gene (10); the so-called acid-sensing ion channels (ASICs), which are mammalian homologues of DEG/ENaCs, have been implicated in mechanical nociception (11); and rare polymorphisms in the calcium channel subunit α2δ3 have been linked to reduced pain phenotypes in humans (6). In addition, dPiezo channels, which are expressed in nociceptive sensory neurons in fly larvae, play an important role in larval mechanical nociception (7), while the human homologue Piezo2 is not only essential for indirectly suppress acute pain (12) and mediating injury-induced tactile pain in mice and humans (13, 14), but also required for gentle touch and proprioception but not mechanical nociception in humans and mice (15, 16). Furthermore, dysregulation of L-VGCCs in the dorsal root ganglia and spinal cord has been shown in neuropathic pain (17), while L-VGCCs have been implicated in modality-specific functions in nociceptive neurons in fly larvae (9, 18, 19).

In response to noxious thermal (heat and cold), mechanical, or chemical stimulation, *Drosophila* larvae display a range of responses: a stereotypic nocifensive response, rolling, which they display when attacked by a parasitic wasp (20); bending of the body (21, 22); forward crawling and backward crawling (23). Noxious information is first processed at specific nociceptive sensory neurons and then in the central nervous system (CNS). The CNS of a fruit fly larva contains approximately 15_,_000 neurons, which is far less than the number of neurons in mammalian brains. This advantage has only recently been utilized owing to the development of several technological advances, including reconstruction of neural circuits at synaptic resolution using transmission electron microscopy (TEM); functional analysis of neurons using genetic tools to selectively target and manipulate individual neuron types; measurement of neural activity through electrophysiological recordings or calcium imaging; and automation of methods for monitoring and categorizing the behaviors of individual larvae.

Here we provide a brief overview of *Drosophila* larva studies that have employed a combination of the above-mentioned technologies to elucidate how ensembles of neurons interact to mediate nociception and trigger a behavior crucial for survival. Studies to date suggest that nociceptive sensory neurons activate a variety of interneurons with diverse patterns of axonal projections [e.g., localized to individual hemi-segments, intersegmental, and ascending to the brain or subesophageal zone (SEZ)], which are associated with the activation of a pair of downstream command-like neurons to drive behavioral output. They also suggest that nociceptive information is integrated by many neurons within the sensorimotor circuitry and that neuromodulators may play a key role in transforming this information to optimize behavioral output. The *Drosophila* larval nociceptive circuitry thus shows promise in providing detailed insights into how the concerted activity of neurons and the circuits in which they are embedded give rise to nociception. We also discuss the similarities between *Drosophila* larval and mammalian nociceptive circuits, as well as directions for future studies to test the extent to which nociceptive circuits are conserved across species.

## 2. *Drosophila* larvae exhibit a wide variety of nociceptive responses to noxious stimuli

For organisms to survive and reproduce, it is essential that they produce appropriate escape responses in the face of life-threatening stimuli. One of the main threats to *Drosophila* larvae in the wild are parasitoid wasps. It is estimated that over 60% of larvae fall prey to parasitization (20). *Drosophila* larvae, which are susceptible to a wide array of noxious stimuli such as mechanical, thermal (heat and cold), chemical, and photic stimuli, exhibit several behavioral responses, such as rolling (a corkscrew-like rotation along the rostrocaudal axis), stopping/freezing, body bending, backward crawling, and forward crawling (20, 23–25). Various methods have been developed to deliver noxious stimuli to *Drosophila* larvae and quantify the response to these stimuli.

### 2.1. Mechanical nociception

Mechanical nociception is assayed using custom-made von Frey filaments calibrated to apply precise forces (30–120 mN) or pressures (above 225 kPa) to larval cuticles (5, 20, 24, 26–28). The observer records the resulting behavior using an ethogram, a catalogue of behaviors including the absence of a response, head withdrawal, stopping, turning, and rolling (24, 28). The behavior evoked depends on the physical properties of the stimuli, such as the site of application and the force applied. With respect to the site of stimulus application, stimulation with a filament applied to the head, middle segments or tail is most likely to elicit freezing followed by backward crawling, rolling or forward crawling, respectively (23, 29). More recently, it has also been demonstrated that the more localized the pressure of the applied stimulation, the greater the likelihood of rolling (19).

### 2.2. Thermal nociception

*Drosophila* larvae, as ectotherms, have a strong preference for a narrow range of temperatures (30) and can be harmed by ambient temperatures. Thermal nociception, i.e., the perception of harmful heat and cold, has been studied in *Drosophila* larvae using various assays (24, 31).

Heat nociception is examined in assays that apply a heat probe to larval abdominal segments 4–6 (24) or use water immersion to increase the temperature over time using a heat plate (25, 32). Larval responses depend on the temperature and mode of heat delivery. For example, in the heat probe assay, temperatures between 38°C and 42°C trigger rolling with long latencies, whereas those between 42°C and 52°C trigger immediate rolling that continues for prolonged periods (24, 25). Above 54°C, larvae rarely withdraw from the probe (25).

Cold nociception is studied using assays that expose larvae to low temperatures controlled by Peltier devices, in which a cold probe is applied focally, or a cold plate applied globally, to the cuticles (31, 33, 34). Cold temperatures below 10°C evoke several distinct stereotyped behaviors, such as head-to-tail contraction, posterior raise, u-shape, and, in some drosophilids, spiracle extension (31, 33, 34).

Since temperature exists as a gradient and clear thresholds can be identified, the thermal assays described above are also used to study complex nociceptive mechanisms such as injury-induced thermal allodynia and hyperalgesia, or to identify the genes involved in thermal nociception (35, 36).

### 2.3. Chemical nociception

Larvae may also be subject to tissue damage from chemical compounds produced by plants [e.g, allyl isothiocyanate (AITC), menthol] (37), and more generally to tissue damage induced by many corrosive agents (e.g., strong acids) (29). Chemical nociception is studied using assays in which the experimenter either applies a concentrated solution of a noxious chemical to the cuticle of a larva, or places the larva in a droplet of the solution, and records the frequencies and latencies of the responses manually (29, 38–40). Previous studies have shown that harmful chemicals evoke a variety of behaviors. For example, whereas menthol and acids trigger rolling, AITC triggers writhing (29, 38–40).

### 2.4. Short-wavelength lights as noxious stimuli

*Drosophila* larvae spend most of their time in dark environments (41, 42). The Bolwig organ, located on the head of the larva, and whose neurons contain photoreceptors that respond to green and blue light, is the primary organ for detecting light in *Drosophila* larvae (42). However, several studies have shown that intense short-wavelength lights (<470 nm) induce head cast followed by crawling behaviors, such as head casting and directional change, mediated by nociceptive sensory neurons in the body wall (22, 43). In addition, intense light has been shown to cause harm or death in some studies (35, 44, 45), suggesting that such lights are nociceptive.

## 3. High-throughput methods to study *Drosophila* larval nociceptive behaviors

High-throughput methods have become increasingly important for studying *Drosophila* larval nocifensive behaviors given their ability to rapidly analyze a large amount of data. Conventional methods for evaluating such behaviors involve manually applying nociceptive stimuli (e.g., a pin prick, a heat or cold probe to a specific area of the abdomen) to individual animals and recording their responses. While these methods have been useful in identifying neurons and genes involved in processing nociceptive sensory inputs and producing nocifensive behaviors, they can be time-consuming and susceptible to individual variability. Recently, new technologies have emerged to allow more objective, high-throughput assessments of behavior in large groups of animals simultaneously.

To meet the need for systematic and efficient delivery of stimuli, stimulation methods have been developed to subject larvae to odors, air currents, vibratory stimuli, heat, cold, and optogenetically manipulated neuronal activity (31, 46, 47). Among thermal stimulation assays, the laser-induced thermal nociception assay using infra-red radiation has been developed to deliver noxious heat (47), while methods employing Peltier-controlled plates have been developed to apply noxious heat or cold to multiple animals (31).

In addition to stimulus delivery methods, there are also several tool available for directly manipulating neural activity in specific neuron types of *Drosophila*. Optogenetics has become one of the most popular tools for activating neurons with high spatial and temporal resolution. Boyden and colleagues (48) reported the first application of the light-gated cation channel, channelrhodopsin-2, which manipulates neural activity by inducing calcium influx at a specific wavelength of light (450–490 nm). To manipulate different neurons separately with distinct optogenetic stimuli, various channelrhodopsins have been discovered or engineered with a wide spectrum of wavelengths (e.g., Chronos, VChR1, C1V1, ReaChR, and Chrimson) (49–52). These channelrhodopsins can depolarized neurons, making it possible to test the gain of function of a specific neuron in the circuit whether activation of specific neurons elicit specific behavior or functional connectivity in the circuit combined with Calcium indicators. In addition, several optogenetic tools such as halorhodopsin, archaerhodopsin-3 and GtACRs, that hyperpolarized neurons have been developed to test the necessity of a neuron for a specific behavior or neural circuit (53–55). Although the optogenetic approach is powerful for high-throuput assays, the optogenetic stimulation is not identical to natural nociceptive sensory stimulation at physiological conditions that typically only activate local subset of sensory neurons.

Other genetic tools that allow for neural manipulation in *Drosophila* include the temperature activated cation channel, dTrpA1 (excitatory) (30, 56–58), the bacterial sodium channel, NaChBac (excitatory) (59), the temperature-sensitive dominant negative form of dynamin Shibire^ts1^(which blocks chemical neurotransmission acutely) (60, 61), the mammalian inward rectifying potassium channel Kir2.1 (inhibitory) (62, 63), and the light chain of tetanus toxin (inhibitory) (64). These tools complement stimulus delivery assays in investigating the neural circuits of various *Drosophila* behaviors, with the caveat that they may stimulate neurons at frequencies and intensities that differ from their natural patterns (which may lead to unconventional behavioral outcomes, e.g., longer/shorter durations for a given behavior, paralysis when all muscles are activated/inhibited). For further details, we refer the reader to a previous review on how to express proteins in specific neurons (65).

To meet the need for large-scale assays, high-throughput data acquisition methods have been developed for recording the responses of multiple larvae. An early study of the roundworm *C. elegans* used the midline of the images to regenerate “eigenworms” (66). This work led to the development of the Multi-Worm Tracker, which is used to analyze the position, speed, and body features of larvae in real time (67). Other larva-tracking software systems, such as MAGAT, FIM, and FIMTrack, have been developed to perform similar tasks (46, 68–70). These systems allow for the efficient and objective measurement of behavior in large groups of larvae.

In recent years, the availability of high-throughput data acquisition methods has led to new approaches to classify behavior patterns. Automated action detection algorithms have been developed by integrating information on body location and shape over time. For example, the Larval Reaction Analysis (LaRA) method was developed to annotate behavioral categories, such as crawling, head casting, hunching, and rolling (47). More recently, machine learning techniques have been used to improve the efficiency of categorizing and detecting behaviors. The Janelia Automatic Animal Behavior Annotator (JAABA) uses a supervised learning method to generate behavioral classifiers for each individual behavior (71, 72). A recent algorithm with pre-trained behavioral classifiers generates behavioral matrices for each larva, allowing simultaneous comprehensive behavior annotation with 6 given behavior classes (73). Unsupervised learning techniques have also been used to develop an unbiased behavioral classification method that focuses on capturing behavior dynamics to cluster them into categories and prevent overfitting of pre-existing behavior patterns, without relying on behaviors defined *a priori* (74, 75).

These automatic high-throughput methodologies, which comprise three consecutive phases in analyzing *Drosophila* larval behavior, allow for more efficient and objective investigation of neurogenetics and behavior (including nocifensive behaviors) in *Drosophila* larvae. By combining these emerging techniques with established approaches, the larval nociceptive system can be studied at the levels of sensory neurons and neural circuits.

## 4. Organization of nociceptive sensory systems in *Drosophila* larvae

**Figure 1:**
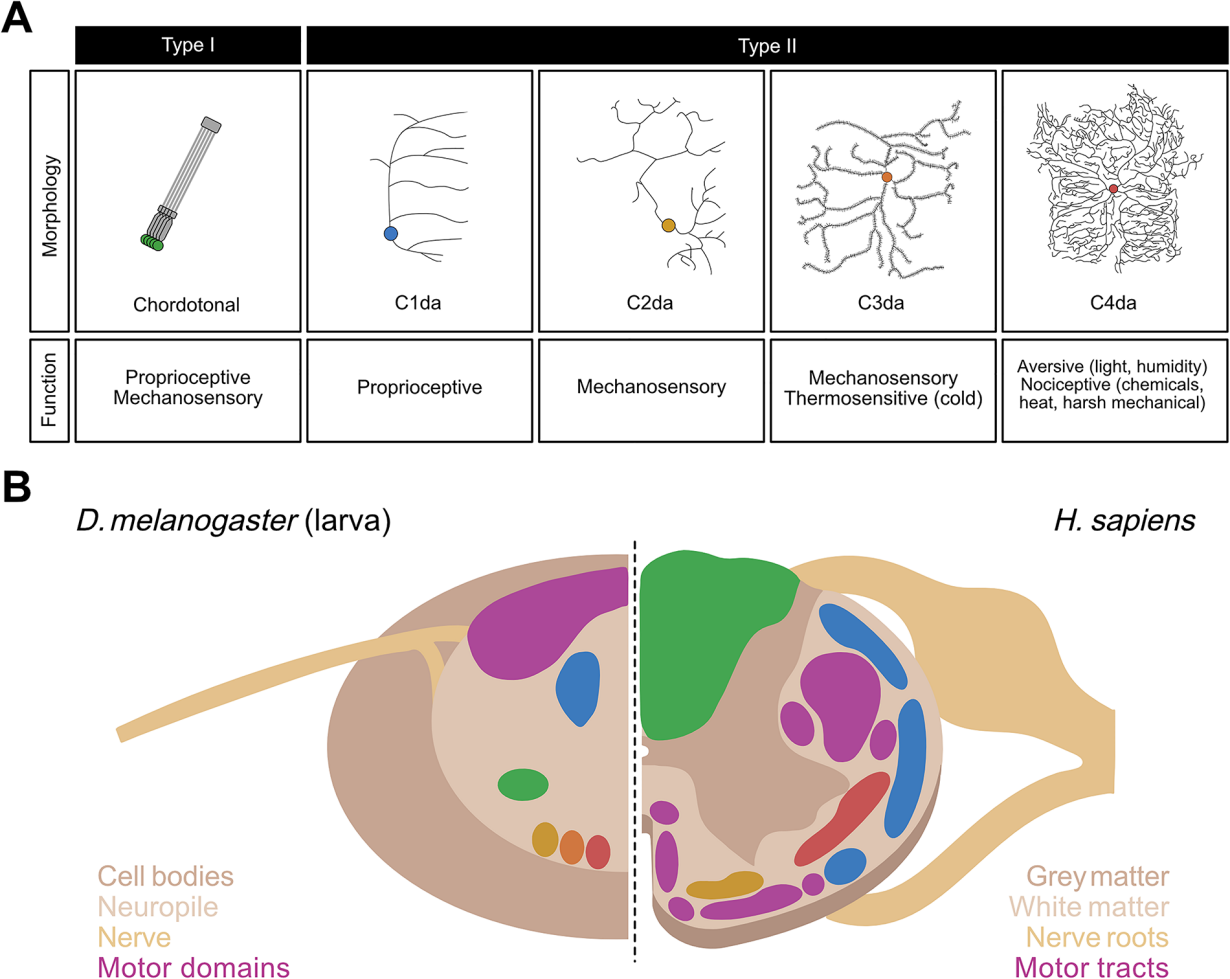
Neurons of the somatosensory system and structural organization of the ventral nerve cord (VNC) in *Drosophila* larvae. (**A**) Morphology and function of the somatosensory neurons in larval *Drosophila melanogaster*. Chordotonal (green), C1da (blue), C2da (yellow), C3da (orange), and C4da (red) neurons are contrasted in terms of their neural morphologies and sensory functions. (**B**) Side-by-side comparison of a sagittal section of the VNC in *D. melanogaster* larvae (left) with that of the spinal cord in *Homo sapiens* (right). Spatially distinct colored regions on the left (green, blue, yellow, orange, and red) represent those occupied by the five classes of Drosophila larval sensory neurons shown in corresponding colors in (**A**). Colored regions on the right represent those in the human spinal cord occupied by sensory neurons whose functions closely correspond to their counterpart neurons in Drosophila larvae, i.e., proprioceptive tracts (blue), fine touch tracts (yellow), tracts carrying mechanosensory and proprioceptive information (green), and nociceptive tracts (red). In both species, motor regions (purple) are spatially segregated from the sensory regions.

*Drosophila* larvae possess sensory systems that detect olfactory, visual, gustatory, proprioceptive, and somatosensory (e.g., mechanosensory, thermosensory, nociceptive) stimuli. Somatosensation and proprioception are mediated by stereotyped segmentally repeated neurons in the body wall of each larval hemisegment (76, 77). These neurons are grouped into four clusters—ventral, ventral', lateral, and dorsal based on the cell body position in the body wall—each of which includes Type I and Type II neurons (77). Type I neurons consist of external sensory (es) neurons whose sensilla are found in small hair-like organs outside of the cuticle, and chordotonal (cho) neurons whose sensory organ, the chordotonal organ, is found under the cuticle (77). In contrast, Type II neurons have multiple dendrites that are located in the epidermis without being embedded in a specialized organ (77, 78) (Figure 1A).

Type II neurons, also known as multidendritic neurons, are subdivided into three broad subtypes: bipolar dendrite neurons, tracheal dendrite neurons, and dendritic arborization (da) neurons. The 15 da neurons per abdominal hemisegment, are further divided into four categories based on the complexity of their dendritic arbor: C1da, C2da, C3da and C4da neurons (78) (Figure 1A). While the dendrites of C1da and C2da neurons have simple branching patterns, C3da neurons show numerous short actin-based processes extending from major branches, and C4da neurons innervate the entire epidermis with complex, space-filling arbors (Figure 1A) (78, 79). The formation of functional sensory circuits requires precise positioning of axons in the CNS. Somatotpic maps are formed by each sensory neuron projecting to highly stereotyped locations in the ventral nerve cord (VNC) (80–83) (Figure 1B). For instance, C4da neurons innervate the ventromedial area of the VNC, whereas cho neurons innervate the ventrolateral area (71, 80, 81) (Figure 1B).

Each subclass of neuron detects a distinct subset of stimuli based on its anatomy and the transduction channels it expresses (Figure 1A) and is sensitive to a wide range of mechanosensory and proprioceptive stimuli (84–87). C1da neurons respond mostly to proprioceptive inputs (88–91); C2da and C3da neurons are both sensitive to gentle mechanosensitive and thermosensitive stimuli (31, 82, 92–94); and C4da and C3da neurons are the most sensitive to nociceptive inputs.

C4da neurons are multimodal sensory neurons whose dendrites tile the body wall of *Drosophila* larvae and detect variety of noxious stimuli, including thermal, mechanical, chemical, and photic stimuli. For example, C4da neurons show strong spiking activity at temperatures above 38°C (24). C4da neurons are sensitive to noxious mechanical stimuli, such as heavy forces and shear stress, but are insensitive to gentle touch (7, 19, 27, 82). In addition, C4da neurons respond to noxious chemical such as AITC and acids and are sensitive to short-wavelength lights (22, 29, 40, 45, 95). Silencing C4da neurons impairs the responses to noxious heat, mechanical stimuli, chemicals, and short-wavelength lights, indicating their importance in nociception (20, 22, 24, 25, 29).

C3da neurons play a critical role for detecting noxious cold in *Drosophila* larvae (31, 33, 34, 36). C3da neurons show increased calcium influx and neuronal spiking as the ambient temperature progressively decreases below 20°C (34, 36). Proper integration of multiple sensory modalities is critical for nocifensive behaviors. For example, neurons dedicated to gentle touch, such as C2da and C3da, appear to facilitate nociception, an effect that may be mediated by the release of sNPF from dorsal pair ilp7 (DP-ilp7) neurons (21). Consistent with this view, silencing these sensory neurons leads to specific impairments in mechanical and chemical nociception (21, 29). As another example, joint activation of chordotonal neurons, which respond to vibration or air currents, and C4da neurons, facilitates nocifensive response, rolling (71). These examples suggest that integration of sensory information plays an important role in properly adjusting nocifensive behaviors in *Drosophila* larvae.

## 5. Circuitry of nociceptive sensory processing

The relatively small CNS of the *Drosophila* larva allows for detailed investigation of neural circuits involved in nociceptive processing, using reconstruction of neurons and neural circuits at synaptic-level resolution from serial section TEM volume images (96, 97). These structural studies of the wiring of neurons (i.e., the connectome), combined with functional studies using various genetic tools to manipulate specific types of neurons and measure downstream neuronal activity or behavior, have identified both excitatory and inhibitory pathways that play a role in nociceptive sensory processing (21, 23, 45, 71, 97–100).

### 5.1. Excitatory networks

**Figure 2:**
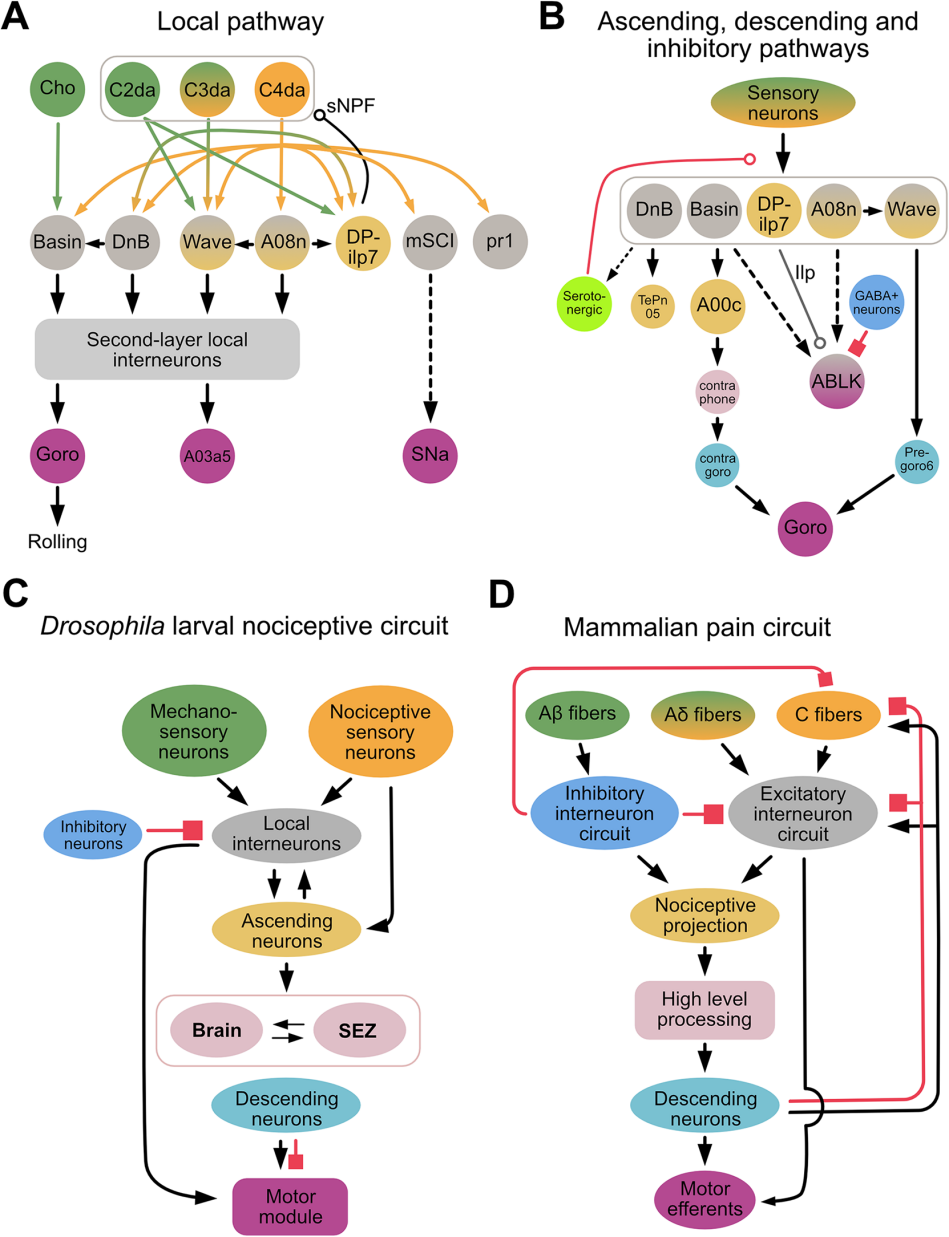
*Drosophila* larval nociceptive circuit. (**A**) A detailed diagram of local excitatory pathways in the *Drosophila* larval nociceptive circuit. Mechanosensory and nociceptive neurons are shown in green and orange, respectively. Local interneurons are shown in gray, and second-layer local interneurons (e.g., A23g, A05q, A02g, T05u, Swallowtail, and A09o) are represented collectively by a gray bracket. The command-like neuron Goro, the pre-motor neuron A03a5, and the motor neuron SNa are shown in purple; these neurons comprise a motor module. (**B**) A diagram showing key neurons comprising the ascending, descending, and inhibitory/modulatory pathways of the *Drosophila* larval nocifensive rolling circuit. Ascending neurons are shown in yellow; neurons in the brain and subesophageal zone (SEZ) in pink; descending neurons in cyan; and inhibitory neurons in blue. In both (**A,B**), bi-colored neurons (e.g., Wave, A08n) send projections locally within the ventral nerve cord (VNC) as well as ascending projections to the brain or SEZ. (Whether ABLK neurons are local interneurons or part of the motor module remains unclear.) Solid and dotted lines indicate direct and indirect connections, respectively. A line terminating in an arrowhead, open circle, or filled square denotes a connection with an excitatory, modulatory, or inhibitory influence, respectively, on the target neuron. In (**B**), smaller neurons represent those identified morphologically from EM reconstruction data but whose biological functions remain unclear. Note that DP-ilp7, which is activated by short-wavelength lights, releases insulin like-peptide (Ilp). The modulatory influence of serotonergic neurons (light green) has thus far only been reported in experience-dependent plasticity during development. (**C,D**). Simplified diagrams of the *Drosophila* larval nociceptive circuit (**C**) and mammalian pain circuit (**D**). In both circuits, multi-modal sensory inputs (green and orange) activate excitatory networks, including integrating layers (gray), ascending projections (yellow), higher-order processing centers (pink), descending pathways (cyan), and motor outputs (purple). In both circuits, several inhibitory neurons (blue) project to various layers.

Several synaptic partners downstream of C4da neurons that play an important role in nocifensive rolling have been identified, including the Basins, A08n, Down & Back [DnB], DP-ilp7, mSCI, pr1, and Wave (21, 23, 71, 97, 99, 100) (Figure 2A). For example, activation of Basins, A08n, DnB, mSCI and pr1 neurons elicits rolling, whereas inhibition of these neurons suppresses rolling. In contrast, activation of DP-ilp7 does not evoke rolling whereas its inhibition is necessary for rolling evoked by noxious mechanical stimulation, suggesting that DP-ilp7 plays a modulatory role (21, 101). Interestingly, the neurons described above receive inputs not only from C4da, but also from other sensory neurons, such as cho, C2da, and C3da. For example, Basins receive inputs from C4da and cho; DnB receives inputs from C4da and C3da (71, 99); and Wave and DP-ilp7 integrate inputs from three types of somatosensory neurons (C2da, C3da, and C4da) (21, 23) (Figure 2A). Whether mSCI and pr1 receive inputs from neurons other than C4da is unknown, but these findings indicate that most second-order interneurons integrate unique combinations of sensory inputs.

The Basins, DnB, A08n, and Wave are indirectly connected *via* local interneurons in the VNC or higher-order brain neurons to the command-like neuron, Goro, whose activation triggers nocifensive rolling (23, 71, 97, 99) (Figure 2B). Activation studies have shown that A00c, a class of ascending neuron that integrates input from Basins across several segments, plays an important role in sending information to the brain (71, 98). Connectome studies have identified other ascending pathways downstream of DnB, as well as connections between A08n and Wave neurons (Figure 2B). How these pathways and connections contribute to nocifensive rolling is currently unclear.

Connectome studies have also identified that Wave and DnB, *via* the local pathway, make direct synaptic contacts with premotor neurons that project to motor modules independent of the Goro pathway (23, 99) (Figure 2B). While the circuit directly downstream of mCSI is not yet fully understood, SNa, a motor neuron further downstream of mCSI, is necessary for mCSI-elicited nocifensive rolling independent of the Goro pathway (102) (Figure 2A).

The function of Wave appears to be segment specific. Activation of Wave in specific abdominal segments 1–3 triggers backward locomotion, a nocifensive response observed when a pinprick stimulus is applied to the head of a larva (23). In contrast, activation of Wave in abdominal segments 4–7 triggers forward locomotion (23). Furthermore, activation of Wave in both the anterior and posterior abdominal segments triggers wiggling and C-bending—responses seen in the initial phases of nocifensive response, rolling—as well as a small number of rolling bouts (23). This segment-specific function of Wave apparently determines what behavior a *Drosophila* larva will exhibit when it receives nociceptive stimulation at different sites along the body (102).

### 5.2. Inhibitory networks

In contrast to excitatory networks, far less is known of how inhibitory networks contribute to nociceptive circuits. One study showed that when larvae were treated with nociceptive chemicals or subjected to optogenetic activation of nociceptive neurons, a serotonergic feedback pathway inhibited nociceptive processing between C4da and their downstream partners (Basins and A08n) (38). Interestingly, abdominal leucokinin (ABLK) neurons, which are downstream of the Basins and A08n, express the serotonin receptor (103), suggesting that serotonin may also affect nociceptive processing *via* leucokinin neurons. Another study demonstrated that GABAergic neurons gate nocifensive rolling by inhibiting ABLK neurons (104) (Figure 2B). Furthermore, a recent Biorxiv paper has purportedly shown that descending inhibitory neurons expressing the neuropeptide drosulfakinin (DSK), a homolog of cholecystokinin (CCK) in mammals, inhibits nocifensive rolling (105). These findings suggest that inhibition of nociceptive information processing in fly larvae may occur *via* several pathways.

## 6. Neuromodulation in the nociceptive circuit

In the *Drosophila* larval nociceptive circuit, both direct synaptic connectivity and neuromodulation play significant roles in shaping behavior. Although DP-ilp7, a second-order neuron downstream of C4da, does not trigger nocifensive rolling on its own, it facilitates rolling through the secretion of short neuropeptide F (sNPF), a homolog of mammalian NPY, which has a dual function in pain perception (i.e., it improves and reduces sensitivity to nociceptive stimulation in different subsets of neurons) (21, 106, 107). The sNPF released by DP-ilp7 binds to the sNPF receptors in C2da, C3da, and C4da neurons, which is specifically necessary to trigger a mechanonocifensive but not thermonocifensive response (21). DP-ilp7 also receives inputs from v'td2 and MIP neurons, which are sensitive to noxious light. When activated by these neurons, however, DP-ilp7 releases Ilp7 (instead of sNPF), which elicits light-evoked head cast behavior. In addition, it has been shown that insulin and tachykinin released upon injury by UV light induces sensitization of nociceptive sensory neurons (35, 108). These findings highlight the potency of neuromodulators in altering how nociceptive sensory neurons process nociceptive stimuli in *Drosophila* larvae (45).

One major factor that affects how the larval nociceptive system processes stimuli is the developmental stage of the animal. Noxious heat or optogenetic activation of C4da neurons elicits rolling in third instar larvae but less rolling in first instar larvae (9, 109, 110). However, harsh mechanical stimuli and optogenetic stimulation of Basin neurons can still trigger rolling in first instar larvae (111), suggesting that their weak rolling in response to noxious heat or optogenetic activation of C4da neurons in first instar larvae, is not due to underdevelopment of their rolling circuit, but rather, is modality-dependent or influenced by other factors. The increased probability of rolling in response to noxious heat as a function of developmental stage may be associated with increased production of ecdysone, a steroidal growth-related hormone required for the initiation of larval pupation (112, 113). A recent study showed that the absence of ecdysone receptor isoforms in C4da neurons led to reduced sensitivity to noxious heat and smaller dendritic arbors (113), suggesting that ecdysone contributes to nociception by altering sensitivity to noxious cues (110).

In addition to endogenous developmental changes, the interaction of a larva with its environment is critical in shaping its nociceptive system. Different environments may present different threats to survival, and larvae must adapt. For example, on the one hand, larvae reared in cold environments become hypervigilant, with several somatosensory neurons, including C3da, showing enhanced responses at temperatures below optimal levels (34). On the other, larvae reared in media containing noxious chemicals become desensitized to noxious stimuli and exhibit less rolling in response to activation of C4da neurons (38). These effects were replicated successfully when optogenetics was used to mimic growth in such noxious environments (34, 38). The desensitization of larvae to noxious stimuli in response to activation of C4da neurons is mediated by serotoninergic feedback at the level of sensory to second-order neurons (38).

## 7. Comparisons between nociceptive systems of *Drosophila* larvae and mammals

The components that enable the detection of noxious stimuli in *Drosophila* larvae are highly homologous to those found in mammals. In mammals, two classes of nerve fibers, the unmyelinated C-fibers and the thinly myelinated A*δ*−fibers, detect noxious stimuli (114). These fibers, which are embedded under the epidermis without specialized organs, rely on the expression of transduction channels at dendrites, and are similar in structure to Type II sensory neurons in *Drosophila* larvae (78, 114). The functions of these fibers in mammals are also similar to those correspondingly assumed by C4da and C3da neurons in *Drosophila* larvae. Specifically, C4da neurons in *Drosophila*, like polymodal C-fibers in mammals, are polymodal nociceptive neurons that detect a wide range of noxious stimuli (20, 22, 24, 29, 38, 43, 114, 115).

The *Drosophila* larval nociceptive circuit and mammalian nociceptive circuits share several similar circuit motifs (Figures 2C,D). In mammals, pain signals are primarily integrated in the dorsal horn within the spinal cord, and then either conveyed to a higher-level processing center (i.e., somatosensory cortex) *via* the spinothalamic tract for further integration and decision-making, or immediately relayed *via* interneurons within the spinal cord to the motor neurons that trigger a rapid response appropriate to the pain (i.e., reflex arc) (116, 117). In *Drosophila* larvae, the nociceptive circuit is organized similarly, in that nociceptive inputs are first processed *via* second-order sensory neurons in the VNC, after which they are conveyed *via* the ascending pathways and local excitatory circuits to the brain and local VNC regions, respectively. At the latter sites, the nociceptive information is integrated with other nociceptive inputs, and then transformed to generate signals that drive motor output (21, 23, 71, 99, 102).

In mammalian nociceptive circuits, low-threshold touch/pressure A*β*-fibers activate inhibitory neurons that gate the high-threshold nociceptive C-fibers (118). Furthermore, a GABAergic circuit projects descending inhibitory inputs from a higher-order processing center (*via* the periaqueductal gray-rostral ventral medulla) to the spinal cord (119, 120). In *Drosophila* larval nociceptive circuits, similar gating motifs filter linear sensory inputs into binary categorical signals to establish a clear threshold for triggering nocifensive rolling (104). The descending pathway that mediates this effect remains unclear, and its identification will be a key step in elucidating the mechanisms underlying such gating of nociceptive inputs.

Lastly, studies of UV-induced tissue damage in *Drosophila* larvae, a model preparation used to investigate peripheral neuropathy in mammals, suggest that several pathways relevant to nociception are conserved. For example, tumor necrosis factor homologue, Eiger which released upon epithelial cell death, increases the sensitivity of C4da neurons to thermal stimuli in *Drosophila* larvae, while tumor necrosis factor, is critical for injury-mediated peripheral neuropathies, such as mechanical allodynia in mammals (35, 121–123). The production of tachykinin, the *Drosophila* homolog of substance P in mammals, disinhibits and sensitizes TrpA1 in C4da neurons following UV-induced tissue damage (124–128) which, in turn, increases the sensitivity of C4da neurons to noxious stimuli and the probability of nocifensive rolling in response to previously innocuous and noxious stimuli (124–126). In mammals, substance P decreases the nociceptive threshold *via* interactions with TRP channels (e.g., TRPV1, TRPA1), suggesting some conservation of the architecture of this peripheral pathway (124, 125, 127, 129, 130). Finally, as in diabetic neuropathy in mammals, ILP signaling is critical for persistence of injury-mediated sensitization, given that loss of ILP2+ neurons or insulin receptors increases rolling after prolonged UV-induced injury (108, 131).

## 8. Conclusion

Fruit fly larvae display various nocifensive behaviors in response to different noxious stimuli. In particular, they display a characteristic nocifensive behavior, rolling, when attacked by a parasitic wasp. Methodological advances in neural circuit reconstruction and high-throughput behavioral analyses have begun to clarify the neural bases of these behaviors. Initial structural analyses of neuronal morphology/connectivity and functional analyses of neural activity identified specialized nociceptive sensory neurons that detect noxious stimuli (mainly C4da neurons); several key interneurons immediately downstream of C4da neurons; and a pair of command-like neurons that drive rolling in an all-or-none fashion. Recent studies have identified inhibitory circuits and neuromodulators that could transform the nociceptive information conveyed *via* the sensory neurons and interneurons to suppress or change the threshold for behavioral output. A key question will be to understand how inhibitory circuits and descending pathways affect rolling at the circuit level. Another will be to determine whether similar circuits and neuromodulators are involved in nocifensive behaviors in vertebrates. The similarities in nociceptive circuits between fly larvae and mammals suggest the potential of using *Drosophila* larvae to illuminate the circuit-level mechanisms of pain in humans.

## Author contributions

J-CB, JZ, and TO wrote the manuscript. All authors contributed to the article and approved the submitted version.
